# Supplementary material for: Predictors of glycemic control, quality of life and diabetes self-management of patients with diabetes mellitus at a tertiary hospital in Ghana
Source: PLoS One. 2025 Jul 3;20(7):e0317075. doi: 10.1371/journal.pone.0317075 (PMC12225827; doi:10.1371/journal.pone.0317075)
Supplement: Attachment — Submitted filename: Glycemic paper.docx [file pone.0317075.s003.docx]

**Running title: Predictors of glycemic control, quality of life and diabetes self-management of patients with diabetes mellitus at a tertiary hospital in Ghana.**

| **General notice:**   - In the formulation of the sentences, it will be noticed that the ‘period’ (full stop) which marks the end of a sentence is most often placed before the in-text references thus distorting the end of a previous statement and beginning of the next statement. - Check on this a correct appropriately for general conformance. - Example Line 64. | | |
| --- | --- | --- |
| Ln 69-70 | - The opening sentence ‘several studies have demonstrated that compare to the general population…’, needs to be revised. It can be understood that authors would want to paraphrase to avoid plagiarism but the sentence restructuring in its current form does not offer clarity. |  |
| Ln 84 | - Consider revising the use of the pronoun ‘his’ as the case group comprises male and female. - Sentence should be more generic to cover both genders. |  |
| Ln 87 | - Revise the use of ‘care’ as it stands vaguely in the context of the sentence. - What does ‘its’ as used refer to? - Check the sentence and revise appropriately. |  |
| Ln 88 | - In what context is ‘overall mean scores’ used? Example, overall mean score for what? - Revise the sentence |  |
| Ln 102 | - The abbreviation for DSM has been presented as DMS |  |
| Ln 147 | - Negatively should read ‘negative’ |  |
| Ln 155 | - Delete ‘the’ preceding ‘patient’s’ |  |
| Ln 162 | - BMI as used must be defined on first use before subsequently presented in abbreviation format. |  |
| Ln 167 | - Provide the manufacturer details for the REDCap software |  |
| Ln 168 | - The first letter of ‘Characteristics’ must not be capitalized |  |
| Ln 187 | - The period should be placed after (Table 1) and not before it as done. |  |
|  | Table 1   - The inclusion of the study site in the heading of the table is not relevant. - Male should be placed in parenthesis - A total number of 360 has been given as part of the table heading but it was realized that the total number presented for HbA1c was 215 - Presenting 215 as the number for HbA1c proves that is a shortfall but no reason has been given in the results section to support this finding. - The percentage estimated from the 215 was used for discussions without recourse to the shortfall. - For characteristic which are presented with / (NHIS/Private Insurance), what do they mean? Does the slash present two items as alternatives? Does the item after the slash represent the point of focus? Authors should check this and correctly. - Examples: No response/Unknown; Foot ulcer/amputation   Number of medications   - What does 0-1 mean in medication delivery? Between 0-1 what did authors count? - Consider using the categories: none, 1 (mono), 2 (dual) and 2+ for multi |  |
| Ln 190 | - Check the spelling for mellitus |  |
| Ln 192-193 | - SGLT-2i and GLP1 were not administered to patients and no record of these were presented on table 1. Delete as part of the footnote. - Why are these presented under the footnote? - Authors should refrain from throwing in variables which hitherto were not assessed just because they intend to include such unexplored data in the discussion. |  |
| Ln 202 | - There is no break point for the previous sentence and as such it follows through into the ensuing sentence. |  |
| Ln 203 | - The percentage estimated for HbA1c (44.7%) is being used without recourse to the fact that it was estimated over 215 and not 360. - 215 represents approximately 60% of the studied population for whom there was evaluable HbA1c data and this must always be factored into the narrative as it has its place in subsequently analysis and clinical extrapolations. |  |
| Ln 209-210 | - There was no need for this statement once again because no information of these drugs was assessed. It can therefore not be added to the presentation just by inclusion to lay foundation to be used for an angle of the discussion as was done in Line 287 - delete |  |
| Ln 224 | - should read logistic and not ‘logistics’ |  |
| Line 231 | - there should be a space after hospitalization and the parenthesis |  |
| Ln 237 | - HbA1c was evaluable for 215 patients. - 145 patients did not have HbA1c results. - What is the measure of good glycaemic control in these 145 patients? - Can FBS be relied upon solely as a guide to good glycaemic control in these patients. |  |
| Line 290 | - the sentence ‘good glycaemic control was observed in 44.7% of patients’ is not a true picture of the findings. - The 44.7% was estimated from 215 and not 360 - 215 represents approximately 60% of the participants. - The 44.7% estimated only reflects information for 60% of the participants and this must be given attention. - In reality, authors missed the point that 40% of the patients are unable to access HbA1c testing. - In the absence of HbA1c results, what will be the best measure of glycaemic control in these patients? |  |
| Ln 312-314 | - Requires revision. |  |
|  | **Discussion**   - Very little discussion was made about the domains of QOL and diabetes self-management score. This takes away immensely from the focus of the study since emphasis was laid on the QOL and DSM. - Emphasis was laid on the independent study variables and their relationship with the dependent variables as assessed yet, not discussion on the domains and scores. - The study did not attempt to evaluate how the covariates influence the QOL domains and DSM scores. - The discussion would have to be revised to include information on which aspects and section of the DSM and QOL questionnaire should be paid attention to in their routine use for long term patient management. |  |
|  | **Conclusion**   - Not suitable for the scope of work done because the analysis fails to explore a relationship between the covariates and the domains of QOL and scores of DSM. This would have thrown much light into areas of the QOL and DSM questionnaires where medical personnel would have to pay much attention during educational sessions for the patients. - As regards QOL, social relationship domain scored the least. The question now arises that what specific steps must be taken to improve this domain? This was not addressed in the discussion but attempts were made to conclude on such albeit not much analysis was conducted on such. - In DSM, glucose management score was the least among the other scores. This observation was not raised for effective discussion to enable readers appreciate the steps that medical personnel will explore to raise the glucose management score to high values and ultimately ensure good quality of life. - The conclusion as it stands does not address any of the concerns raised by way of arguments in the background section. |  |
