# Supplementary material for: Predictors of glycemic control, quality of life and diabetes self-management of patients with diabetes mellitus at a tertiary hospital in Ghana
Source: PLoS One. 2025 Jul 3;20(7):e0317075. doi: 10.1371/journal.pone.0317075 (PMC12225827; doi:10.1371/journal.pone.0317075)
Supplement: Attachment — Submitted filename: Response to Reviewers.docx [file pone.0317075.s006.docx]

**Response to reviewers**

**The authors appreciate the extensive review done and are happy to incorporate the corrections, recommendations and suggestions to improve this manuscript.**

Reviewer #1:

Line 87: delete the 4th word :and"

**Response: Thanks for the correction. The change has been appropriately effected in the manuscript. (Line 88)**

Line 96: Start a new sentence with the 5th word :However"

**Response: Thanks for the correction. The change has been appropriately effected in the manuscript. (Line 97)**

Line 147: Delete the 2nd word "and"

**Response: Thanks for the correction. The change has been appropriately effected in the manuscript. (Line 153)**

Line 157: Change the 8th word "include" to "included"

**Response: Thanks for the correction. The change has been appropriately effected in the manuscript. (Line 163)**

Line 168: Indicate which type of statistical software you used for the analysis here

**Response: Thanks for the correction. The change has been appropriately effected in the manuscript. (Line 174)**

Line 279: Start as a new sentence

**Response: Thanks for the correction. The change has been appropriately effected in the manuscript. (Line 305)**

Line 287: Start as a new sentence

**Response: Thanks for the correction. The change has been appropriately effected in the manuscript. (Line 313)**

Lines 304-306: What reasons can you assign to your finding that insulin use is associated with poor glycaemic control in the study population?

**Response: The reasons as proposed in the manuscript (Lines 332-345) include:**

**-Most participants (95% in the current study being Type 2 DM) require insulin only when their glycemic control is already bad. This is a form of association by indication.**

**-Another reason may be inertia on the part of doctors and patients to initiate insulin in patients who require insulin due to poor glycemic control. Thus delayed insulin initiation would result in worsening of glycemic control.**

**-Barriers to insulin use which may contribute to poor glycemic control were not fully elucidated in the current study. These include hypoglycemia and fear of hypoglycemia, weight gain, needle anxiety, insufficient education, lack of access to insulin and poor insulin storage. The need for glucose monitoring and insulin dose adjustment may also be a challenge as this present study shows the weakest domain of DSM to be glucose management.**

**Reviewer #2:**

The study underscores a holistic approach to the real world management of DM as experienced

by both healthcare providers and patients. The study finding add to scientific knowledge, The manuscript was well written and structurally organized.

However the authors need to check table 1, the frequency for the female was missing (line 188, p8)

**Response: Thanks for the correction. The change has been appropriately effected in the manuscript. (Line 196)**

Reviewer #3:

This work looked at a very salient area in diabetic management. However, I have some comments to help improve the quality of the manuscript. I am attaching my comments since it exceeds the word limit.

| **General notice:**   - In the formulation of the sentences, it will be noticed that the ‘period’ (full stop) which marks the end of a sentence is most often placed before the in-text references thus distorting the end of a previous statement and beginning of the next statement. - Check on this a correct appropriately for general conformance. - Example Line 64.   **Response: Thanks for the correction. The changes have been appropriately effected in the entire manuscript.** | | |
| --- | --- | --- |
| Ln 69-70 | - The opening sentence ‘several studies have demonstrated that compare to the general population…’, needs to be revised. It can be understood that authors would want to paraphrase to avoid plagiarism but the sentence restructuring in its current form does not offer clarity.   **Response: Thanks for the correction. The sentence has been restructured for clarity. (Line 70-72)** |  |
| Ln 84 | - Consider revising the use of the pronoun ‘his’ as the case group comprises male and female. - Sentence should be more generic to cover both genders.   **Response: Thanks for the correction. The change has been appropriately effected in the manuscript. (Line 85)** |  |
| Ln 87 | - Revise the use of ‘care’ as it stands vaguely in the context of the sentence. - What does ‘its’ as used refer to? - Check the sentence and revise appropriately.   **Response: Thanks for the correction. The sentence has been restructured for clarity. (Line 88)** |  |
| Ln 88 | - In what context is ‘overall mean scores’ used? Example, overall mean score for what? - Revise the sentence   **Response: Thanks for the correction. The sentence has been revised appropriately. (Line 89)** |  |
| Ln 102 | - The abbreviation for DSM has been presented as DMS   **Response: Thanks for the correction. The change has been appropriately effected in the manuscript. (Line 104)** |  |
| Ln 147 | - Negatively should read ‘negative’   **Response: Thanks for the correction. The change has been appropriately effected in the manuscript. (Line 153)** |  |
| Ln 155 | - Delete ‘the’ preceding ‘patient’s’   **Response: Thanks for the correction. The change has been appropriately effected in the manuscript. (Line 161-162)** |  |
| Ln 162 | - BMI as used must be defined on first use before subsequently presented in abbreviation format.   **Response: Thanks for the correction. The change has been appropriately effected in the manuscript. (Line 169)** |  |
| Ln 167 | - Provide the manufacturer details for the REDCap software   **Response: Thanks. The manufacturer details have been provided. (Line 173-174)** |  |
| Ln 168 | - The first letter of ‘Characteristics’ must not be capitalized   **Response: Thanks for the correction. The change has been appropriately effected in the manuscript. (Line 175)** |  |
| Ln 187 | - The period should be placed after (Table 1) and not before it as done.   **Response: Thanks for the correction. The change has been appropriately effected in the manuscript. (Line 195)** |  |
|  | Table 1   - The inclusion of the study site in the heading of the table is not relevant.   **Response: Thanks for the correction. The change has been appropriately effected in the manuscript. (Line 196)**   - Male should be placed in parenthesis   **Response: Thanks for the recommendation. Both male and female have been provided in the table and formatted uniformly. (Line 196)**   - A total number of 360 has been given as part of the table heading but it was realized that the total number presented for HbA1c was 215 - Presenting 215 as the number for HbA1c proves that is a shortfall but no reason has been given in the results section to support this finding. - The percentage estimated from the 215 was used for discussions without recourse to the shortfall.   **Response: Thanks for this salient observation. The fact that some members of the study population did not have evaluable HbA1c was discussed as part of the limitations of this study with specific reason adduced. (Line 423-425)**  **The reason for the short fall is further given in the results section and subsequent discussions have been put in context. (Lines 42-44, 203-204, 214-216, 241, 252 and 316-323)**   - For characteristic which are presented with / (NHIS/Private Insurance), what do they mean? Does the slash present two items as alternatives? Does the item after the slash represent the point of focus? Authors should check this and correctly. - Examples: No response/Unknown; Foot ulcer/amputation   **Response: Thanks for the recommendation. The slash represents alternatives. This has been corrected appropriately in all the tables. (Lines 196, 252, 258, 264 and 270)**  Number of medications   - What does 0-1 mean in medication delivery? Between 0-1 what did authors count? - Consider using the categories: none, 1 (mono), 2 (dual) and 2+ for multi   **Response: Thanks for the recommendation. This has been corrected appropriately in all the tables. (Lines 196, 252, 258, 264 and 270)** |  |
| Ln 190 | - Check the spelling for mellitus   **Response: Thanks for the correction. The change has been appropriately effected in the manuscript. (Line 198)** |  |
| Ln 192-193 | - SGLT-2i and GLP1 were not administered to patients and no record of these were presented on table 1. Delete as part of the footnote. - Why are these presented under the footnote? - Authors should refrain from throwing in variables which hitherto were not assessed just because they intend to include such unexplored data in the discussion.   **Response: Thanks for the observation and we apologize for the misrepresentation. The authors investigated specifically the use of these medications among participants and recorded zero usage. It is unfortunate that the data presented on the Table 1 had SGLT-2i and GLP-1 analogues omitted because the frequency and percentage was 0. This omission has been appropriately corrected in Table 1. (Line 196)** |  |
| Ln 202 | - There is no break point for the previous sentence and as such it follows through into the ensuing sentence.   **Response: Thanks for the correction. The change has been appropriately effected in the manuscript. (Line 214)** |  |
| Ln 203 | - The percentage estimated for HbA1c (44.7%) is being used without recourse to the fact that it was estimated over 215 and not 360. - 215 represents approximately 60% of the studied population for whom there was evaluable HbA1c data and this must always be factored into the narrative as it has its place in subsequently analysis and clinical extrapolations.   **Response: Thanks for this salient observation. The fact that some members of the study population did not have evaluable HbA1c was discussed as part of the limitations of this study with specific reason adduced. (Line 423-425)**  **The reason for the short fall is further given in the results section and subsequent discussions have been put in context. (Lines 42-44, 203-204, 214-216, 241, 252 and 316-323)** |  |
| Ln 209-210 | - There was no need for this statement once again because no information of these drugs was assessed. It can therefore not be added to the presentation just by inclusion to lay foundation to be used for an angle of the discussion as was done in Line 287 - Delete   **Response: Thanks for the observation and we apologize sincerely for the misrepresentation. The authors investigated specifically the use of these medications among participants and recorded zero usage. It is unfortunate that the data presented on the Table 1 had SGLT-2i and GLP-1 analogues omitted because the frequency and percentage was 0. This omission has been appropriately corrected in Table 1. (Line 196)** |  |
| Ln 224 | - should read logistic and not ‘logistics’   **Response: Thanks for the correction. The change has been appropriately effected in the manuscript. (Line 239)** |  |
| Line 231 | - there should be a space after hospitalization and the parenthesis   **Response: Thanks for the correction. The change has been appropriately effected in the manuscript. (Line 247)** |  |
| Ln 237 | - HbA1c was evaluable for 215 patients. - 145 patients did not have HbA1c results. - What is the measure of good glycaemic control in these 145 patients? - Can FBS be relied upon solely as a guide to good glycaemic control in these patients.   **Response: Thanks for this salient observation. Mean FBG in mmol/L of participants with HbA1c (n=215) is 9.1±4.9 and of participants without HbA1c (n=145) is 8.8 ±4.7 (p=0.541) (Line 203-204)**  **In the present study, there was no statistical difference between the mean FBG of participants with HbA1c and those without HbA1c hence FBG could be relied upon as a guide to good glycemic control in these patients. (Line 320-322)** |  |
| Line 290 | - the sentence ‘good glycaemic control was observed in 44.7% of patients’ is not a true picture of the findings. - The 44.7% was estimated from 215 and not 360 - 215 represents approximately 60% of the participants. - The 44.7% estimated only reflects information for 60% of the participants and this must be given attention. - In reality, authors missed the point that 40% of the patients are unable to access HbA1c testing. - In the absence of HbA1c results, what will be the best measure of glycaemic control in these patients?   **Response: Thanks for this salient observation. Subsequent discussions have been put in context. (Lines 42-44, 203-204, 214-216, 241, 252 and 316-323)**  **Alternatives to assessing glycemic control when HbA1c is not accessible may include using FBG, post prandial glucose and self-monitoring of blood glucose. (Line 322-323)** |  |
| Ln 312-314 | - Requires revision.   **Response: Thanks for the recommendation. The discussion has been appropriately revised to read: “Income levels also played a significant role in determining QOL. In this case, better QOL was noted if respondents earned between two and ten times the minimum wage (GH₵ 541 to 2700, that is, USD 46 to 230) compared to household incomes less than twice the minimum wage. It appears that, these respondents had sufficient resources to cope with their condition. This finding is consistent with past studies conducted in Africa, yielding the conclusion that at precariously low income levels, QOL might generally be adversely affected due to psychological distress [46, 47].” (Lines 350-355)** |  |
|  | **Discussion**   - Very little discussion was made about the domains of QOL and diabetes self-management score. This takes away immensely from the focus of the study since emphasis was laid on the QOL and DSM. - Emphasis was laid on the independent study variables and their relationship with the dependent variables as assessed yet, not discussion on the domains and scores. - The study did not attempt to evaluate how the covariates influence the QOL domains and DSM scores. - The discussion would have to be revised to include information on which aspects and section of the DSM and QOL questionnaire should be paid attention to in their routine use for long term patient management.     **Response: Thanks for the recommendation. The methodology and discussion has been appropriately expanded to include the domains of QOL and DSM scores. (Lines 141-146, 182-183, 346, 365-383, 386-387, 391-393, 398-400, 410-421 and 433)**  **Further analysis has been done to evaluate how the covariates influence the QOL domains and DSM scores. (Lines 182-183, 274-293, 295 and 297)**  **Critical aspects of the QOL and DSM questionnaire for routine long term patient management have been highlighted. (Lines 141-146, 372-373, 379-380 and 417-421)** |  |
|  | **Conclusion**   - Not suitable for the scope of work done because the analysis fails to explore a relationship between the covariates and the domains of QOL and scores of DSM. This would have thrown much light into areas of the QOL and DSM questionnaires where medical personnel would have to pay much attention during educational sessions for the patients. - As regards QOL, social relationship domain scored the least. The question now arises that what specific steps must be taken to improve this domain? This was not addressed in the discussion but attempts were made to conclude on such albeit not much analysis was conducted on such. - In DSM, glucose management score was the least among the other scores. This observation was not raised for effective discussion to enable readers appreciate the steps that medical personnel will explore to raise the glucose management score to high values and ultimately ensure good quality of life. - The conclusion as it stands does not address any of the concerns raised by way of arguments in the background section.   **Response: Thanks for the recommendation. The methodology and discussion has been appropriately expanded to include the domains of QOL and DSM scores. (Lines 141-146, 182-183, 346, 365-383, 386-387, 391-393, 398-400, 410-421 and 433)**  **Further analysis has been done to evaluate how the covariates influence the QOL domains and DSM scores. (Lines 182-183, 274-293, 295 and 297)**  **Further discussions have particularly focused on social relationships domain of QOL and glucose management domain of DSM exploring avenues for improvement. (Lines 373-383 and 410-421)**  **The conclusion therefore lies within the scope of the work done and addresses arguments raised in the background.** |  |

**Kindly note the following changes to the reference list.**

**Ten new references have been added during the revision process:**

36. Ketema EB, Kibret KT. Correlation of fasting and postprandial plasma glucose with HbA1c in assessing glycemic control; systematic review and meta-analysis. Arch Public Heal [Internet]. 2015 Sep 25 [cited 2025 Mar 13];73(1):43. Available from: <https://pmc.ncbi.nlm.nih.gov/articles/PMC4582842/>

43. Bayked EM, Kahissay MH, Workneh BD. Barriers and facilitators to insulin treatment: a phenomenological inquiry. J Pharm Policy Pract [Internet]. 2022 Dec 1 [cited 2025 Mar 12];15(1):1–11. Available from: <https://joppp.biomedcentral.com/articles/10.1186/s40545-022-00441-z>

44. Chen R, Aamir AH, Feroz Amin M, Bunnag P, Chan SP, Guo L, et al. Barriers to the Use of Insulin Therapy and Potential Solutions: A Narrative Review of Perspectives from the Asia–Pacific Region. Diabetes Ther [Internet]. 2024 Jun 1 [cited 2025 Mar 12];15(6):1261–77. Available from: <https://link.springer.com/article/10.1007/s13300-024-01568-9>

51. Marengoni A, Angleman S, Melis R, Mangialasche F, Karp A, Garmen A, et al. Aging with multimorbidity: A systematic review of the literature. Ageing Res Rev. 2011 Sep 1;10(4):430–9.

52. Uhing A, Williams JS, Garacci E, Egede LE. Gender Differences in the Relationship between Social Support and Strain and Mortality among a National Sample of Adults. J Behav Med [Internet]. 2021 Oct 1 [cited 2025 Mar 15];44(5):673. Available from: <https://pmc.ncbi.nlm.nih.gov/articles/PMC8484032/>

53. Stephenson KR, Pickworth C, Jones PS. Gender differences in the association between sexual satisfaction and quality of life. Sex Relatsh Ther [Internet]. 2024 [cited 2025 Mar 15];39(2):301–22. Available from: <https://www.tandfonline.com/doi/abs/10.1080/14681994.2021.2004309>

54. Ramanan VV, Singh SK. A study on alcohol use and its related health and social problems in rural Puducherry, India. J Fam Med Prim Care [Internet]. 2016 [cited 2025 Mar 15];5(4):804. Available from: <https://pmc.ncbi.nlm.nih.gov/articles/PMC5353818/>

55. Ramesh S, Tomy C, Nair RR, Olickal JJ, Joseph JK, Thankappan KR. Correlation of self-management and social support with quality of life in patients with chronic kidney disease undergoing hemodialysis: A cross-sectional study from Kerala, India. Clin Epidemiol Glob Heal. 2024 Sep 1;29:101731.

56. Tannor EK, Norman BR, Adusei KK, Sarfo FS, Davids MR, Bedu-Addo G. Quality of life among patients with moderate to advanced chronic kidney disease in Ghana - A single centre study. BMC Nephrol [Internet]. 2019 Apr 8 [cited 2025 Mar 15];20(1):1–10. Available from: <https://bmcnephrol.biomedcentral.com/articles/10.1186/s12882-019-1316-z>

59. Anene-Okeke CG, Mosanya AU, Osakwe O. Knowledge, Practice, and Barriers Regarding Self-monitoring of Blood Glucose Among Patients with Type 2 Diabetes Mellitus in Enugu State, Nigeria. J Diabetol [Internet]. 2021 Jul [cited 2025 Mar 16];12(3):310–8. Available from: <https://journals.lww.com/jodb/fulltext/2021/12030/knowledge,_practice,_and_barriers_regarding.12.aspx>
