# Supplementary material for: Predictors of glycemic control, quality of life and diabetes self-management of patients with diabetes mellitus at a tertiary hospital in Ghana
Source: PLoS One. 2025 Jul 3;20(7):e0317075. doi: 10.1371/journal.pone.0317075 (PMC12225827; doi:10.1371/journal.pone.0317075)
Supplement: Attachment — Submitted filename: Reviewer Comemnts Predictors_Glycemic.docx [file pone.0317075.s005.docx]

**Comments: Predictors of glycaemic control**

| Line | Comment |
| --- | --- |
| 39 | Multivariable logistics should read multivariable logistic |
| 62 | Mentions the overall prevalence of DM as 6.46% with no year reference and this was compared with 0.2% in 1964. Which year relative to 1964 is being evaluated? |
| 131 | The ‘q’ term for the Fisher formula was not defined. |
| 172 | It indicates that poor DSM was defined as DSM score <6 making reference to Schmitt et al., (1996).  A critical evaluation of the scoring guide indicates that the use of a cut-off score of <6 to detect persons with suboptimal or non-adherent self-management behaviours is not recommended. The statement is as refers below:  **NOT RECOMMENDED**: *To detect persons with suboptimal or non-adherent self-management behaviours, a cut-off score of < 6 on the total scale has been utilized in some studies – This is not recommended! This criterion most likely originates from an abstract publication (see ref. no. 4 above) reporting on a study in which 226 German PWD were categorised according to their DSMQ total scores using median split. (“the median split yielded a cut-off score of ≈ 6*  *on the DSMQ scale ranging from 0 to 10.”) By design, this score split the sample into two halves, reflecting that a cut-off score of < 6 would identify about half of the sample with suboptimal self-management behaviour, thus not being suitable for detecting clinical cases.*  Was the choice of a cut-off score <6 arrived at based on a median split for the cohort of patients being evaluated? |
| 230 | In Line 158, authors indicated a percentage transformation of scale scores ranging from 0 to 100.   1. How was a score <6 chosen as a cut-off for poor DSM on a 0 to 100 scale?   The DSM scores stated in this section ranged from 0 to 10. No score exceeded 10 thus defying the transformation score of 0 to 100 stated by authors. |
| 236 | The sum score is 10 and not 100 as expected from authors statement in Line 158 |
| 240-241 | The statement reads: ‘*patients on only oral DM medications (aOR 2.14; 95% CI 1.19-3.88, p=0.012) were more likely to have good glycemic control compared to those on insulin when HbA1c (n=215) was used as a measure of glycemic control (Table 4a).*   1. From table 4a, under DM medications used, the covariates evaluated were ‘Dual or Multi’ and ‘no insulin’. Authors evaluated ‘no insulin’ but in the statement were presenting information on insulin use. This is contradictory and must be re-evaluated. 2. The aOR of 2.14 was an adjusted OR for ‘no insulin’ in the table but this was presented as an output for oral medications which had no results outcome in the adjusted analysis column. 3. The analysis from the table shows that patients who are not on insulin had good glycaemic control compared to others. |
| 242-243 | The statement reads: ‘*FBG as a measure of glycemic control yielded similar results (aOR 1.82; 95% CI 1.12-2.96,p=0.017)*’   1. This statement was made drawing inference from the HbA1c statement in Line 240. It is worthy to note that the interpretation error made in Line 240 likewise runs through the assertion for this statement. 2. From the table Dual/Multi and No Insulin as covariates had outputs under the univariate and adjusted models. 3. From the output, no insulin is associated with good glycaemic control as measured by FBG in the univariate and adjusted models and the opposite for Dual/Multi medications. 4. It cannot therefore be the case or suggestion that oral medications were associated with good glycaemic control based on FBG because the analysis does not support that assertion. |
| 248 | Period is placed before the parenthesis. |
| 295 | Table 7  Based on the foregoing arguments on the use of 6 as a score cut-off, further analysis based on score cut-off ≈6 requires re-evaluation. |
| 302-303 | The statement reads: ‘*on only oral DM medications to be associated with good glycemic control as compared with patients on insulin*’   1. This assertion is not supported by the evidence from the analysis shown on Tables 4a and 4b. The tables evaluated ‘no insulin’ and there was no indication from the analysis that oral medications were associated with good glycaemic control. 2. Take note, no analysis from the results section supports oral medication over insulin. |
| 303 | Statements like, ‘*Socio-economic factors such as urban residence and household income were protective of poor QOL’,* can be expressed in a better way in the discussion than the persistent use of qualifiers such as ‘poor QOL’ in the analysis.  What does protective of poor QOL mean in this scenario? |
| 304 | *‘Good overall DSM was also associated with good QOL’*  statements like this can be expressed better to give better meaning than the format of applying qualifiers. |
| 314 | Statement was made about SGLT-2 inhibitors and GLP-1 agonists and how beneficial they are in diabetic patient populations. The fact remains that none of the patients evaluated were on SGLT-2 inhibitors and GLP-1 agonists and there was no way the benefits of these drugs could be assessed in this study.  This statement is therefore not relevant.  Given the fact that no patient were on these drugs and no analysis on benefit was conducted, the statement on gap in access cannot be supported in this study. |
| 321 | *‘FBG could be relied upon as a guide to good…’*   1. FBG is already in use as a diagnostic criterion for diabetes care and management. 2. The fact that authors could did not observed any statistical difference in mean FBG for patients with HbA1c and those without HbA1c is not scientific and clinical proof enough to suggest and propose FBG as a guide to good glycaemic control. |
| 323 | Self-monitoring of blood glucose was stated as an alternative to assessing glycaemic control when HbA1c is no accessible.  Fact is, self-monitoring of blood glucose will still be limited to FBG estimation and as such does not provide any varied alternative to FBG which was listed in addition to post prandial glucose.  The section needs revision |
| 328 | *The differences in glycemic control across studies and countries may be due to differences in treatment strategies, access to specialized care, DSM education and socio-cultural factors*  This too general of a statement for which critical analysis was not conducted in this study. One would want to know the specific item lines associated with treatment strategies, access to specialized care and socio-cultural factors mediating differences in glycaemic control. |
| 331 | The statement reads: ‘*Our study showed that being on only oral DM medications was associated with good glycemic control’*  This assertion is not supported by the analysis as indicated in comments regarding medication above. This assertion must be revised based on corrections and deductions drawn from the analysis |
| 338 | The statement reads: *Our experience seems to conform to insulin use being associated with poor glycemic control by indication.*  As the analysis will show, insulin use was not assessed in this study. What was assessed was ‘no insulin’ and the interpretation on this must be well reviewed by authors. |
| 340-341 | The statement reads: *Barriers to insulin use which may contribute to poor glycemic control were not fully elucidated in the current study*  The study never assessed barriers to insulin use and as such cannot suggest or ascribe reasons to that which was not evaluated in the discussion. |
| 411 | The conclusion as it stands does not fully address the true findings from the study and the potential lessons and implications for diabetic patient management. Consider revising. |
