# Supplementary material for: Predictors of glycemic control, quality of life and diabetes self-management of patients with diabetes mellitus at a tertiary hospital in Ghana
Source: PLoS One. 2025 Jul 3;20(7):e0317075. doi: 10.1371/journal.pone.0317075 (PMC12225827; doi:10.1371/journal.pone.0317075)
Supplement: Attachment — Submitted filename: Response to Reviewers_R2.docx [file pone.0317075.s007.docx]

**Response to reviewers**

**Thank you very much for the extensive review done to improve this manuscript. The authors are happy to incorporate the corrections, recommendations and suggestions.**

Reviewer #3:

The authors tried to respond to the queries I raised in the first revision. However, there still remain some few comments that needs to be answered. This has been attached as a separate document.

| Line | Comment |
| --- | --- |
| 39 | Multivariable logistics should read multivariable logistic  **Response: The change has been effected. (Line 39)** |
| 62 | Mentions the overall prevalence of DM as 6.46% with no year reference and this was compared with 0.2% in 1964. Which year relative to 1964 is being evaluated?  **Response: The statement has been revised to include the year being referenced. “according to a systematic review and meta-analysis by Asamoah-Boaheng et al in 2019”. (Line 64)** |
| 131 | The ‘q’ term for the Fisher formula was not defined.  **Response: All the terms for the Fisher’s formula have been defined. “q = (1- p)”. (Line 135)** |
| 172 | It indicates that poor DSM was defined as DSM score <6 making reference to Schmitt et al., (1996).    A critical evaluation of the scoring guide indicates that the use of a cut-off score of <6 to detect persons with suboptimal or non-adherent self-management behaviours is not recommended. The statement is as refers below:  **NOT RECOMMENDED**: *To detect persons with suboptimal or non-adherent self-management behaviours, a cut-off score of < 6 on the total scale has been utilized in some studies – This is not recommended! This criterion most likely originates from an abstract publication (see ref. no. 4 above) reporting on a study in which 226 German PWD were categorised according to their DSMQ total scores using median split. (“the median split yielded a cut-off score of ≈ 6*  *on the DSMQ scale ranging from 0 to 10.”) By design, this score split the sample into two halves, reflecting that a cut-off score of < 6 would identify about half of the sample with suboptimal self-management behaviour, thus not being suitable for detecting clinical cases.*  Was the choice of a cut-off score <6 arrived at based on a median split for the cohort of patients being evaluated?  **Response: The choice of cut-off score <6 was not based on a median split for the current cohort of patients being evaluated but was based on the abstract referenced in your comment. The investigators deliberated on the options of using -1 SD as cut-off as was used for QOL in the present study. However, the previously documented cut-off in literature of <6 was settled on without keen observation of its limitations. The authors have therefore established a cut-off using the current population distribution characteristic at -1 SD from the mean. This new analysis output has been used in the relevant tables of results. (Line 174)** |
| 230 | In Line 158, authors indicated a percentage transformation of scale scores ranging from 0 to 100.   1. How was a score <6 chosen as a cut-off for poor DSM on a 0 to 100 scale?   The DSM scores stated in this section ranged from 0 to 10. No score exceeded 10 thus defying the transformation score of 0 to 100 stated by authors.  **Response: The actual scale scores range from 0 to 10. The authors have corrected the error in Line 158 which should have read “Sums of item scores are calculated to give scale scores and then converted into a scale that ranges from 0 to 10 (raw score/theoretical maximum score *10).” (Lines 159-161)** |
| 236 | The sum score is 10 and not 100 as expected from authors statement in Line 158  **Response: The authors have corrected the error in Line 158 which should have read “Sums of item scores are calculated to give scale scores and then converted into a scale that ranges from 0 to 10 (raw score/theoretical maximum score *10).” (Lines 159-161)** |
| 240-241 | The statement reads: ‘*patients on only oral DM medications (aOR 2.14; 95% CI 1.19-3.88, p=0.012) were more likely to have good glycemic control compared to those on insulin when HbA1c (n=215) was used as a measure of glycemic control (Table 4a).*   1. From table 4a, under DM medications used, the covariates evaluated were ‘Dual or Multi’ and ‘no insulin’. Authors evaluated ‘no insulin’ but in the statement were presenting information on insulin use. This is contradictory and must be re-evaluated. 2. The aOR of 2.14 was an adjusted OR for ‘no insulin’ in the table but this was presented as an output for oral medications which had no results outcome in the adjusted analysis column. 3. The analysis from the table shows that patients who are not on insulin had good glycaemic control compared to others.   **Response: The authors recognize the incompleteness of the labelling of variables in the (Tables 4a to 6) under DM medications as against the actual analysis done which leads to the misinterpretation of the results. These analysis outputs should have been reported separately, expanded under two different headings i.e “Number of DM medications used” and “DM medications used” (ref Table 1). Under Number of DM medications used “Dual or multi” was compared to “None or mono” while under DM medications used “No insulin” was compared to “Insulin”. This correction has been made in all the relevant tables to give an accurate representation of the analysis performed. (Lines 254, 260, 266 and 272)**  **Also, in the abstract, results and subsequent discussion, the description of “not being on insulin” has been appropriately used instead of the description “only oral medications”. (Lines 42, 242, 306, 333 and 433)** |
| 242-243 | The statement reads: ‘*FBG as a measure of glycemic control yielded similar results (aOR 1.82; 95% CI 1.12-2.96,p=0.017)*’   1. This statement was made drawing inference from the HbA1c statement in Line 240. It is worthy to note that the interpretation error made in Line 240 likewise runs through the assertion for this statement. 2. From the table Dual/Multi and No Insulin as covariates had outputs under the univariate and adjusted models. 3. From the output, no insulin is associated with good glycaemic control as measured by FBG in the univariate and adjusted models and the opposite for Dual/Multi medications. 4. It cannot therefore be the case or suggestion that oral medications were associated with good glycaemic control based on FBG because the analysis does not support that assertion.   **Response: Same as above. This correction has been made in all the relevant tables to give an accurate representation of the analysis performed, and in the subsequent discussions. (Lines 254, 260, 266 and 272) and (Lines 42, 242, 306, 333 and 433)** |
| 248 | Period is placed before the parenthesis.  **Response: This has been corrected. (Line 250)** |
| 295 | Table 7  Based on the foregoing arguments on the use of 6 as a score cut-off, further analysis based on score cut-off ≈6 requires re-evaluation.  **Response: The authors have recognized this and as such subsequent analysis have been based on an established cut-off using the current population distribution characteristic at -1 SD from the mean. The new analysis output has been used in all the relevant tables of results.**  **(Lines 254, 260, 266 and 272)** |
| 302-303 | The statement reads: ‘*on only oral DM medications to be associated with good glycemic control as compared with patients on insulin*’   1. This assertion is not supported by the evidence from the analysis shown on Tables 4a and 4b. The tables evaluated ‘no insulin’ and there was no indication from the analysis that oral medications were associated with good glycaemic control. 2. Take note, no analysis from the results section supports oral medication over insulin.   **Response: Same as above. This correction has been made in all the relevant tables to give an accurate representation of the analysis performed, and in the subsequent discussions. (Lines 254, 260, 266 and 272) and (Lines 42, 242, 306, 333 and 433)** |
| 303 | Statements like, ‘*Socio-economic factors such as urban residence and household income were protective of poor QOL’,* can be expressed in a better way in the discussion than the persistent use of qualifiers such as ‘poor QOL’ in the analysis.  What does protective of poor QOL mean in this scenario?  **Response: This statement and other such persistent use of qualifiers have been revised in the discussion and relevant sections of the manuscript. (Line 307)** |
| 304 | *‘Good overall DSM was also associated with good QOL’* statements like this can be expressed better to give better meaning than the format of applying qualifiers.  **Response: This statement has been revised as recommended. The analysis has been done with poor DSM (< -1 SD) to be the covariate with the output to suggest its association with worse QOL appropriately. This correction has been made in all the tables and discussed appropriately without the need for too many qualifiers. (Line 308 ) and (Lines 254, 260, 266, 272, 299 and 301)** |
| 314 | Statement was made about SGLT-2 inhibitors and GLP-1 agonists and how beneficial they are in diabetic patient populations. The fact remains that none of the patients evaluated were on SGLT-2 inhibitors and GLP-1 agonists and there was no way the benefits of these drugs could be assessed in this study.  This statement is therefore not relevant.  Given the fact that no patient were on these drugs and no analysis on benefit was conducted, the statement on gap in access cannot be supported in this study.  **Response: The two statements have been omitted.** |
| 321 | *‘FBG could be relied upon as a guide to good…’*   1. FBG is already in use as a diagnostic criterion for diabetes care and management. 2. The fact that authors could did not observed any statistical difference in mean FBG for patients with HbA1c and those without HbA1c is not scientific and clinical proof enough to suggest and propose FBG as a guide to good glycaemic control.   **Response: The authors have omitted this statement.** |
| 323 | Self-monitoring of blood glucose was stated as an alternative to assessing glycaemic control when HbA1c is no accessible.  Fact is, self-monitoring of blood glucose will still be limited to FBG estimation and as such does not provide any varied alternative to FBG which was listed in addition to post prandial glucose.  The section needs revision  **Response: The authors have revised this section. Self-monitoring of blood glucose provides a dynamic blood glucose profile rather than a single snapshot of blood glucose. It is therefore useful in resource-poor settings where ambulatory continuous glucose monitoring systems are unavailable. (Line 324,324)** |
| 328 | *The differences in glycemic control across studies and countries may be due to differences in treatment strategies, access to specialized care, DSM education and socio-cultural factors*  This too general of a statement for which critical analysis was not conducted in this study. One would want to know the specific item lines associated with treatment strategies, access to specialized care and socio-cultural factors mediating differences in glycaemic control.  **Response: This section has been revised and focuses on only the studies referenced. “The differences in glycemic control across these studies may be due to the study designs: single-center versus multi-center and primary care versus tertiary care settings. The definition of glycemic control whether based on FBG or glycated hemoglobin also varies across the studies.” (Lines 329-332)** |
| 331 | The statement reads: ‘*Our study showed that being on only oral DM medications was associated with good glycemic control’*  This assertion is not supported by the analysis as indicated in comments regarding medication above. This assertion must be revised based on corrections and deductions drawn from the analysis  **Response: Same as above. This correction has been made in all the relevant tables to give an accurate representation of the analysis performed, and in the subsequent discussions. (Lines 254, 260, 266 and 272) and (Lines 42, 242, 306, 333 and 433)** |
| 338 | The statement reads: *Our experience seems to conform to insulin use being associated with poor glycemic control by indication.*  As the analysis will show, insulin use was not assessed in this study. What was assessed was ‘no insulin’ and the interpretation on this must be well reviewed by authors.  **Response: Same as above. This correction has been made in all the relevant tables to give an accurate representation of the analysis performed, and in the subsequent discussions. (Lines 254, 260, 266 and 272) and (Lines 42, 242, 306, 333 and 433)** |
| 340-341 | The statement reads: *Barriers to insulin use which may contribute to poor glycemic control were not fully elucidated in the current study*  The study never assessed barriers to insulin use and as such cannot suggest or ascribe reasons to that which was not evaluated in the discussion.  **Response: This statement has been omitted.** |
| 411 | The conclusion as it stands does not fully address the true findings from the study and the potential lessons and implications for diabetic patient management. Consider revising.  **Response: The conclusion has been revised to fully address the true findings from the study. Lessons and implications for management of patients with diabetes have also been summarized. (Lines 50-54 and 430-442)** |

**Kindly note the following changes to the reference list.**

**Reference 43 and 44 have been removed during the revision process.**

43. Bayked EM, Kahissay MH, Workneh BD. Barriers and facilitators to insulin treatment: a phenomenological inquiry. J Pharm Policy Pract [Internet]. 2022 Dec 1 [cited 2025 Mar 12];15(1):1–11. Available from: <https://joppp.biomedcentral.com/articles/10.1186/s40545-022-00441-z>

44. Chen R, Aamir AH, Feroz Amin M, Bunnag P, Chan SP, Guo L, et al. Barriers to the Use of Insulin Therapy and Potential Solutions: A Narrative Review of Perspectives from the Asia–Pacific Region. Diabetes Ther [Internet]. 2024 Jun 1 [cited 2025 Mar 12];15(6):1261–77. Available from: <https://link.springer.com/article/10.1007/s13300-024-01568-9>

**One new reference 57 has been added.**

57. Suara SB, Siassi F, Saaka M, Rahimi Foroshani A, Sotoudeh G. Association between Carbohydrate Quality Index and general and abdominal obesity in women: a cross-sectional study from Ghana. *BMJ Open*. 2019;9(12):e033038. doi:10.1136/BMJOPEN-2019-033038
